# Elucidation of a Unique Pattern and the Role of Carbohydrate Binding Module of an Alginate Lyase

**DOI:** 10.3390/md18010032

**Published:** 2019-12-30

**Authors:** Fu Hu, Benwei Zhu, Qian Li, Heng Yin, Yun Sun, Zhong Yao, Dengming Ming

**Affiliations:** 1 College of Food Science and Light Industry, Nanjing Tech University, Nanjing 211816, China; hufu@njtech.edu.cn (F.H.); njlq@njtech.edu.cn (Q.L.); sunyun_food@njtech.edu.cn (Y.S.); yaozhong@njtech.edu.cn (Z.Y.); 2 Dalian Institute of Chemical Physics, Chinese Academy of Sciences, CAS, Dalian 116023, China; yinheng@dicp.ac.cn; 3 College of Biotechnology and Pharmaceutical Engineering, Nanjing Tech University, Nanjing 211816, China

**Keywords:** alginate lyase, trisaccharide preparation, degrading pattern, unique mechanism, oligosaccharides

## Abstract

Alginate oligosaccharides with different degrees of polymerization (DPs) possess diverse physiological activities. Therefore, in recent years, increasing attention has been drawn to the use of enzymes for the preparation of alginate oligosaccharides for food and industrial applications. Previously, we identified and characterized a novel bifunctional alginate lyase Aly7A, which can specifically release trisaccharide from three different substrate types with a unique degradation pattern. Herein, we investigated its degradation pattern by modular truncation and molecular docking. The results suggested that Aly7A adopted a unique action mode towards different substrates with the substrate chain sliding into the binding pocket of the catalytic domain to position the next trisaccharide for cleavage. Deletion of the Aly7A carbohydrate binding module (CBM) domain resulted in a complex distribution of degradation products and no preference for trisaccharide formation, indicating that the CBM may act as a “controller” during the trisaccharide release process. This study further testifies CBM as a regulator of product distribution and provides new insights into well-defined generation of alginate oligosaccharides with associated CBMs.

## 1. Introduction

Alginate is a linear anionic polysaccharide that is the major component of the cell walls of brown algae. It is composed of the monomeric units 1,4-linked β-d-mannuronate (M) and its C5 epimer, α-l-guluronate (G) [1]. These units are arranged in block structures comprising homopolymeric G blocks (PolyG), M blocks (PolyM), and heteropolymeric MG or GM blocks (PolyMG or PolyGM) [2]. Alginate oligosaccharides are depolymerized degradation products of alginate that are produced by physicochemical methods or through the action of alginate lyases. They have attracted increasing attention in recent years owing to their diverse biological activities, including anti-tumor and anti-viral properties, and their ability to regulate immune functions. Furthermore, alginate oligosaccharides are widely utilized in the food, agricultural, and pharmaceutical industries as additives and therapeutic agents [3,4,5,6,7,8]. For example, they are used to lower blood sugar and lipids, and have shown considerable potential as a treatment for cystic fibrosis [9,10]. Currently, physical methods and chemical treatments are the main methods used to produce alginate oligosaccharides with a low degree of polymerization (DP). However, it is almost impossible to scale up the production of alginate oligosaccharides using these methods owing to low product yields and a requirement for expensive equipment and time-consuming processes.

Alginate lyases, which are members of the polysaccharide lyase (PL) family, catalyze alginate degradation via a β-elimination mechanism, releasing unsaturated oligosaccharides containing a uronic acid moiety at their non-reducing termini [11], and provide an alternative approach for the preparation of alginate oligosaccharides. To date, alginate lyases have been isolated from a variety of organisms including marine mollusks [12,13,14], seaweeds [14], several marine bacteria and fungi [15,16,17,18,19,20], bacteriophages, and viruses [21,22]. In addition to their application in the production of alginate oligosaccharides [23,24,25,26,27], alginate lyases have been utilized in the elucidation of alginate fine structures [28,29,30], the preparation of red and brown algal protoplasts [31], and the treatment of cystic fibrosis in combination with other chemotherapeutics [32,33,34]. However, the applications of alginate lyases are currently limited by high application cost and low application value. Alginate lyases can be classified into two subgroups based on their substrate specificities: mannuronate lyases (EC 4.2.2.3) and guluronate lyases (EC 4.2.2.11), which preferentially hydrolyze M- and G-rich alginates, respectively. Enzymes exhibiting specificity for G or M blocks are designated as monofunctional, whereas those specific for MG blocks are termed bifunctional [35]. Bifunctional lyases, which can produce alginate oligosaccharides with specific DPs, have shown considerable potential in industrial applications. Therefore, continued screening to identify alternative alginate lyases is vital to meet industrial demand and fulfill their potential applications.

Alginate lyases are modular proteins that contain non-catalytic domain and catalytic domain [36]. The catalytic domain contributes to recognizing and degrading substrates, while carbohydrate binding module (CBM), as a non-catalytic domain, often plays a special role in binding substrates, controlling products and affecting thermo-stability [36,37,38]. For instance, CBM can enhance the substrate preference and enzyme activity [38], change the distribution of oligosaccharide products [36], and increase the protein thermo-stability [37].

Previously, we purified and characterized a new alginate lyase Aly7A from *Vibrio* sp. W13 [39]. Aly7A contains carbohydrate binding module (CBM) and catalytic domain based on bioinformatics analysis. Intriguingly, Aly7A specifically produces trisaccharide as mainly productions. We speculate that CBM may affect the product preference of full-length enzyme. In this study, we characterized its substrate degradation pattern and elucidated the role for its carbohydrate binding module (CBM).

## 2. Results

### 2.1. Sequence analysis of Aly7A

**Figure 1:**
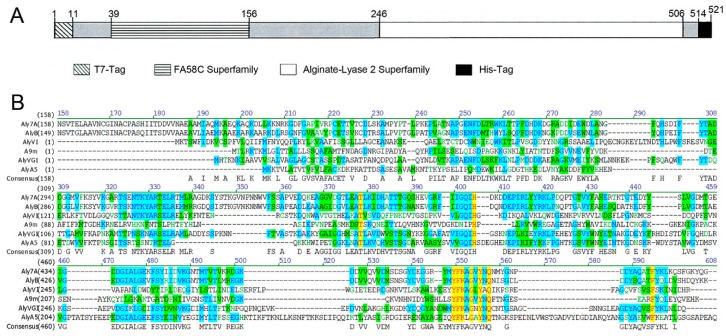
Domain and sequence analysis of Aly7A. (**A**) Schematic domain diagram of Aly7A.Four parts in the 521-residue recombinant enzyme Aly7A: T7-tag (1–11), the N-terminal carbohydrate binding module (39–156), the C-terminal catalytic domains (246–506), and His-tag (514–521). The domains were predicted and analyzed by Simple Modular Architecture Research Tool (SMART). (**B**) Aly7A and related alginate lyase sequences alignments. Aly7A from *Vibrio sp.* W13 (AIY22655) [39], AlyB from *Vibrio splendidus*OU02 (PDB: 5ZU5) [41], A9m from *Vibrio* sp. JAM-A9m (BAH79131) [42], AlyVI from *Vibrio* sp. QY101 (AAP45155) [43], AlyVGI from *Vibrio halioticoli*IAM14596T (AAF22512), AlyA from *Zobellia galactanivorans* DsijT (CAZ98266) [44].

As shown in Figure 1A, recombinant Aly7A contained two structural domains: a putative carbohydrate binding module (CBM) and an alginate lyase-2 catalytic domain (Aly7A-CD) that was predicted to possess substrate-binding and catalytic activities, respectively. A multiple sequence alignment analysis revealed that the catalytic and substrate binding sites of Aly7A and other PL-7 family members were highly conserved (Figure 1B). Obviously, the conserved regions “YFKA/VGN/VY” and “QIH” were conserved in these alginate lyases, which were necessary for catalysis and binding of substrates [40]. Full-length Aly7A and a catalytic domain derivative (Aly7A-CD; residues 246–506) were successfully expressed and purified for further characterization studies. The purified Aly7A and Aly7A-CD were analysed by SDS-PAGE (as shown in Figure S1). The molecular weight of Aly7A is between 50 kDa and 60 kDa, and the molecular weight of Aly7A-CD is approximately 30 kDa, both of which are consistent with their theoretical molecular weight. 

### 2.2. Analysis of Aly7A and Aly7A-CD Degradation Products

**Figure 2:**
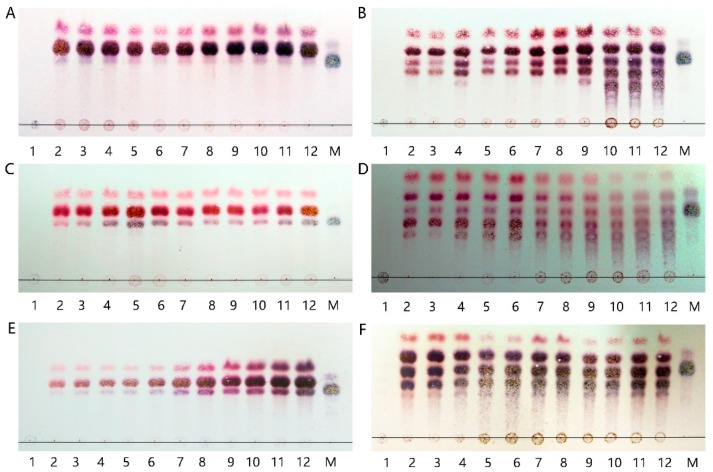
TLC analysis of the degradation products of Aly7A (**A**,**C**,**E**) and Aly7A-CD (**B**,**D**,**F**) towards alginate sodium, polyM, and polyG. Lane 112: degradation products of substrates for 0 min, 5 min, 10 min, 15 min, 30 min, 1 h, 2 h, 4 h, 6 h, 12 h, 24 h, and 48 h. Lane M: tetrasaccharide.

The patterns of hydrolysis products released from three different alginate substrates following their incubation with Aly7A and Aly7A-CD for different time periods (0–48 h) were analyzed by TLC (Figure 2). The hydrolysis products released from sodium alginate, polyM, and polyG substrates by the action of full-length Aly7A are shown in Figure 2A,C,E respectively. In the initial reaction stages, the alginate polysaccharide substrate incubated with full-length Aly7A rapidly decreased with a concomitant increase of oligosaccharides with a DP of 2–4. This indicated that Aly7A may possibly function as an endo-type lyase. However, a substantial amount of trisaccharides also appeared within a short time after the reaction was initiated, which is significantly different from the hydrolysis patterns reported for other endo-type enzymes. This suggests that Aly7A may adopt a unique degradation pattern towards different substrates compared with other alginate lyases previously characterized.

The hydrolysis products released from sodium alginate, polyM, and polyG substrates by the action of Aly7A-CD are shown in Figure 2B,D,F, respectively. In all three cases, the distributions of degradation products were more complex and showed no preference for trisaccharide formation. Otherwise, the catalytic activities of Aly7A towards alginate were 15,718 U/mg, 35,524 U/mg and 18,770 U/mg, while the catalytic activities of Aly7A-CD were 14,010 U/mg, 29,321 U/mg and 22,941 U/mg. Therefore, we concluded that the CBM in full-length Aly7A mainly aids the formation of trisaccharides instead of increasing the enzymatic activity of the catalytic domain [41].

**Figure 3:**
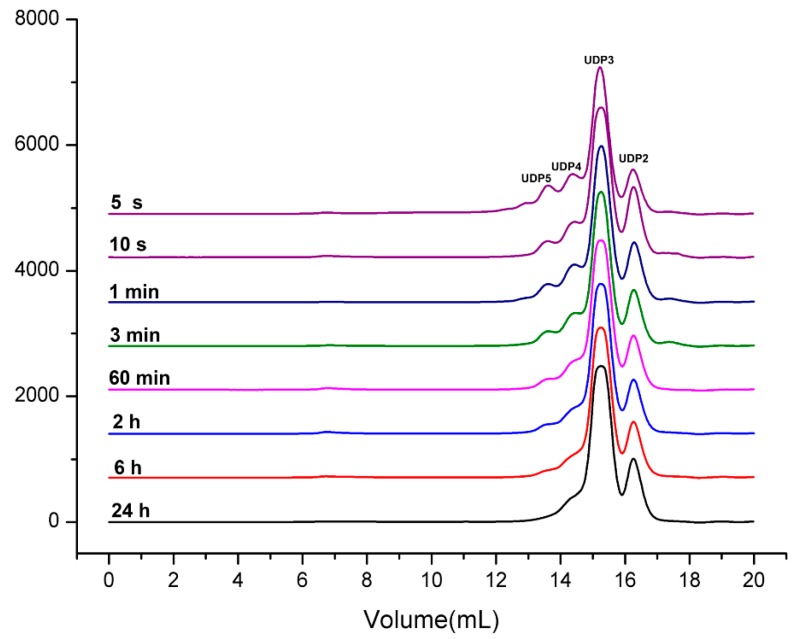
FPLC analysis of Aly7A degrading products towards alginate for different times. Aly7A degraded alginate for 5 s, 10 s, 1 min, 3 min, 60 h, 2 h, 6 h, 24 h. The degradation products were separated by Superdex Peptide 10/300 GL column with 200 mM NH_4_HCO_3_. FPLC monitored oligosaccharide products with UV-detector at 235 nm.

The degradation products of Aly7A at different time points were separated and monitored by FPLC with Superdex Peptide 10/300 GL column at 235 nm after being treated. As shown in Figure 3, at the initial stage of the degradation, oligosaccharides with higher Dps appeared with large fraction of trisaccharides. As the reaction progresses, the composition of the degradation products becomes simpler and the oligosaccharides with higher Dps such as pentasaccharide and tetrasaccharide were further hydrolyzed into oligosaccharides with lower Dps such as trisaccharide and disaccharide.

**Figure 4:**
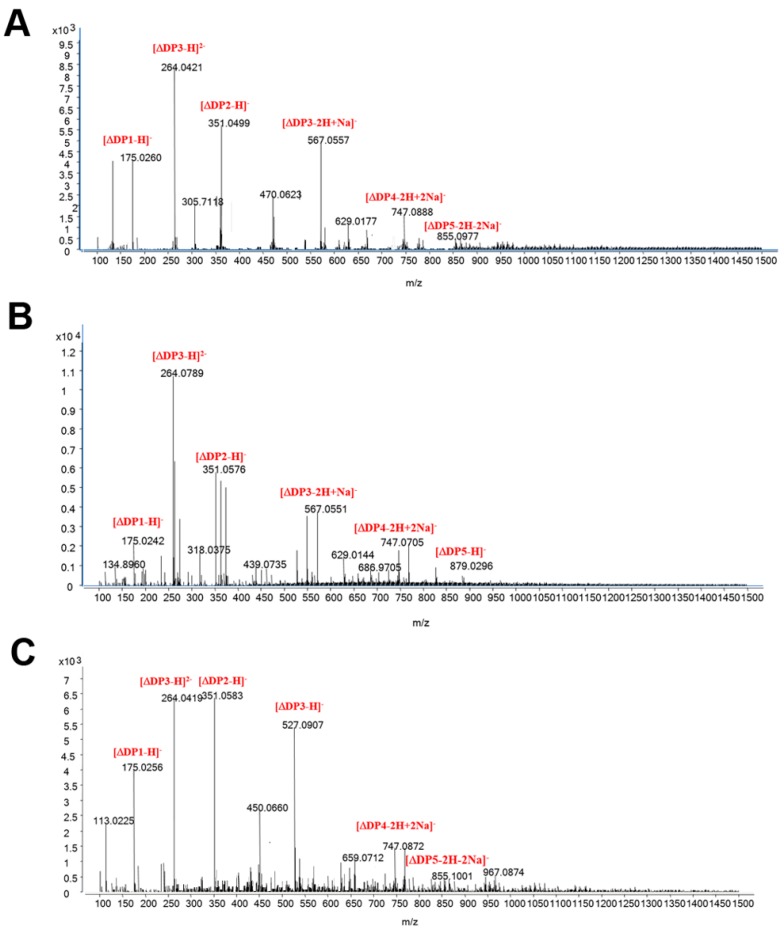
ESI-MS analysis of degradation products of Aly7A-CD with (**A**) alginate sodium, (**B**) polyM, (**C**) polyG as substrates. The degradation products of Aly7A-CD were purified and concentrated. The mass-to-charge ratios (*m*/*z*) of oligosaccharide products were detected by using ESI-MS.

We then used ESI-MS to further assay the compositions of the degradation products of Aly7A-CD (Figure 4). Consistent with our TLC results, a significant proportion of the Aly7A degradation products were trisaccharide with a small fraction of disaccharides and tetrasaccharide as previously reported [39]. In contrast, the Aly7A-CD degradation products included oligosaccharides with a range of DPs (Figure 4A–C). Additionally, the degradation products of Aly7A and Aly7A-CD were also separated and monitored by FPLC with Superdex Peptide 10/300 GL column at 235 nm (Figure S2). The FPLC result further proved the ESI-MS and TLC result.

### 2.3. Affinity Electrophoresis of CBM

**Figure 5:**
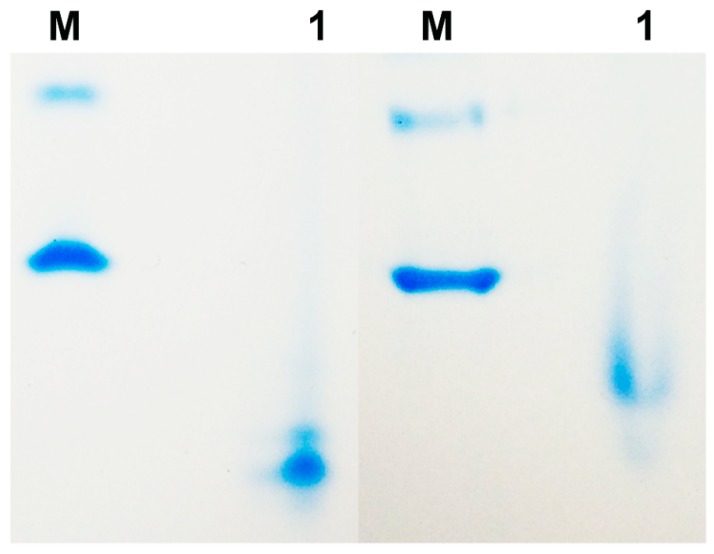
The affinity electrophoresis of CBM in native gel with and without 0.2% of alginate (Left part, the native gel without 0.2% of alginate; right part, the native gel with 0.2% of alginate). Lane M: Albumin from bovine serum (BSA).

The interaction of the CBM towards the alginate was investigated by affinity electrophoresis. As shown in Figure 5, the CBM move more slowly in the native gel than that in the control gel and the result indicated that an interaction between CBM and substrate did, in fact, occur.

### 2.4. Molecular Modeling and Docking Analysis of Aly7A

**Figure 6:**
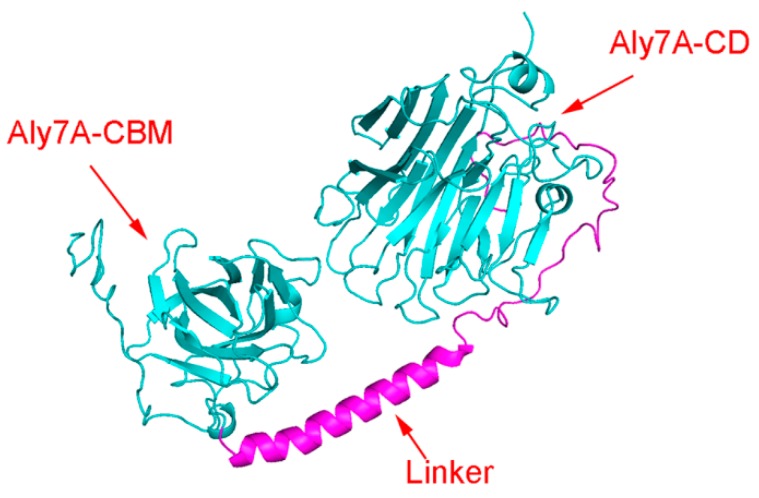
Stereo view of three-dimensional structures of Aly7A. Homologous modeling simulated the protein structure of Aly7A. Aly7A contained two domains which were linked by a sequence named “Linker”. There domains were called Aly7A-CBM and Aly7A-CD based on conserved domain sequence analysis, separately.

A three-dimensional model of full-length Aly7A was constructed based on a homologous structure of AlyB from *Vibrio splendidus*OU02 (PDB ID: 5ZU5) using PHYRE2. This protein has the 70% similarity to Aly7A. The protein model was successfully constructed with 100% confidence as AlyB and Aly7A are closely related and share a high sequence similarity. Furthermore, Aly7A is predicted to have the same β-jelly roll folding pattern as AlyB. As shown in Figure 6, an N-terminal CBM domain (left part) and a C-terminal catalytic domain (right part) constitute the predicted Aly7A structure. The CBM domain (Pro_39_–Gln_156_) exhibits a typical CBM fold which contains eight β-strands with a small α-helical section between the first and second strand. We suppose that Aly7A-CBM may belong to CBM32 based on sequence alignment and homologous modeling. The C-terminal catalytic domain (Asn_246_–Gln_506_) shows an obvious β-sandwich jelly roll fold. It consists of two layers of antiparallel and stacked β-strands. The linker is not shown in the domain analysis (Figure 1A) but found in the molecular modeling as a helix (Figure 6). These secondary structures of Aly7A are similar to AlyB [41].

**Figure 7:**
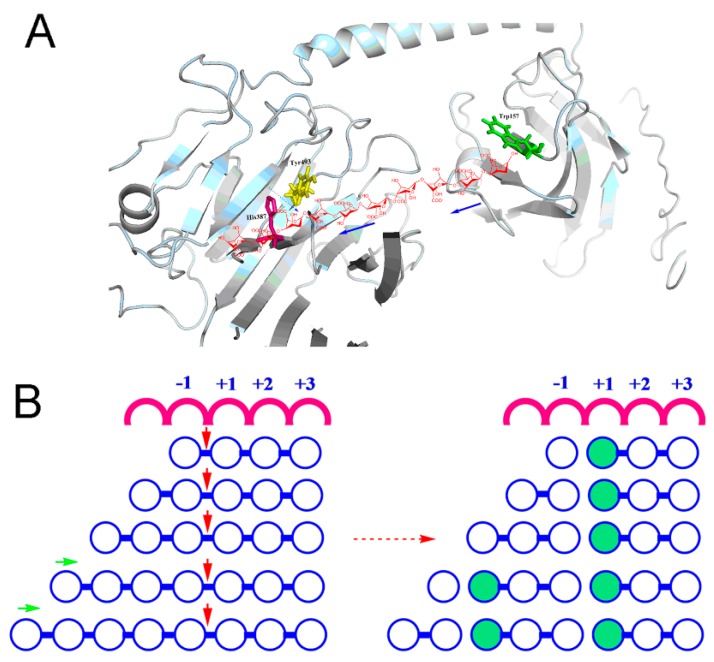
(**A**) Stereo view of the combination of the tunnel-shaped active site of Aly7A with alginate oligosaccharide. (**B**) Putative action pattern of Aly7A towards different substrates. The solid red arrow represents prior cleavage and the green arrow represents the direction of the action.

The Aly7A catalytic domain was initially defined to be residues 263–506 based on multiple sequence alignments with reported PL7 structures. However, a truncated enzyme exhibited no activity towards the three substrate types (data not shown). Asn_246_–Phe_263_ region may also be part of the catalytic domain which is suggested by AlyB (PDB: 5ZU5) full-length structure (shown in purple in Figure 6). It is possible that this short region plays an essential role in maintaining the activity of Aly7A. The key residues for substrate recognition were predicted to be residues Gln_385_, His_387_ and Tyr_493_. These highly conserved residues were involved in the interaction between substrates and protein (Figure 7). From our sequence align and docking analysis results, we propose that Tyr_493_ plays an essential role in recognizing and cleaving the bond between the subsites −1 and +1, while the residues Gln_385_ and His_387_ form hydrogen bonds with the carboxyl groups in subsites +2 and +3.

The homologous modeling structure and docking analysis indicate that the active site of Aly7A is distributed within two flexible loops (Figure 7A). We suppose that these flexible loops may adjust the substrate binding pocket to assist the active site in catalyzing different substrates. This result is similar with active site in AlyB [41]. It has been speculated that the flexibility of these loops is essential for both substrate recognition and binding. Both M and G block substrates can bind to the active site, indicating that Aly7A has broad substrate specificity. Our docking results in combination with the interaction analysis between the catalytic residues and the alginate oligosaccharide substrate suggest that the subsites +1, +2, and +3 at the reducing end act as a product release site. The trisaccharide product is released after a cleavage between –1 and +1 (Figure 7B). We concluded that the substrate chain could then slide into the binding pocket to position the next trisaccharide for cleavage (Figure 7A). As Lyu et al. have reported, a putative role for the alpha-helical linker is to keep the CBM and CD at an appropriately mutual location and distance between the two binding pockets to ensure that the alginate substrate enters the catalytic cavity in a precise manner during this processive movement [41]. Therefore, we propose that the CBM and the alpha helix linker, in combination, act as a “controller” that is responsible for product distribution and ensures a consistent release of trisaccharide products.

## 3. Discussion

Previously, we identified and characterized a novel bifunctional alginate lyase Aly7A, which can specifically release trisaccharides from three different substrate types with a unique degradation pattern. It contains two structural domains: a putative carbohydrate binding module (CBM) and a catalytic domain (Aly7A-CD). We suppose that CBM may affect the full-length enzyme Aly7A specifically produces trisaccharides. To investigate this assumption, the function of the catalytic domain in isolation, Aly7A-CD, a truncated form of the enzyme was also expressed, purified and characterized. Comparing full-length enzyme Aly7A and truncation Aly7A-CD, Aly7A showed activities towards both polyM and polyG substrates, indicating that it is a bifunctional alginate lyase. Furthermore, our kinetics results indicate that Aly7A had the strongest affinity to polyMG substrates. Aly7A-CD also possessed high activities towards alginate, polyM and polyG and, like full-length Aly7A, exhibited the highest catalytic efficiency towards M block and hybrid MG substrates rather than G block substrates (Table S1).

Intriguingly, the products of Aly7A and Aly7A-CD toward alginate, polyM and polyG were vastly different (Figure 2 and Figure S2). The distribution of the degradation products released by Aly7A-CD was substantially more complex compared with the full-length Aly7A and included DPs of 2–5. In addition, it showed no preference for trisaccharide cleavage. Therefore, we concluded that the CBM in combination with the alpha-helix linker in Aly7A function to ensure the predominant production of trisaccharides rather than merely increasing the enzymatic activity of the catalytic domain [41].

To date, most characterized enzymes of the PL7 family are endolytic alginate lyases, which are able to hydrolyze their alginate substrates into oligosaccharides and release a series of oligomeric products with different DPs [15,16,18,19,20]. The majority of the products were oligosaccharides with low DPs of 2–5. However, we revealed that Aly7A specifically produces trisaccharides, which is a significantly different activity from that of other endo-type enzymes that have been previously characterized [39]. This result indicates that Aly7A has adopted a unique degradation pattern towards a range of substrates. Intriguingly, AlyA5 from *Zobellia galactanivorans* can degrade alginate into disaccharides in an exolytic manner, and therefore, shares some similar characteristics with Aly7A [45]. In addition, the AlyB from *Vibrio splendidus*OU02 also showed a similar catalytic mode with Aly7A towards alginate. When alginate was used as substrate, the AlyB could degrade alginate into several oligosaccharides in an exolytic manner, of which trisaccharides as main production.

To elucidate the unique degradation pattern of Aly7A, we performed homology modeling and a molecular docking analysis. These indicated that the residues Gln_385_, His_387_ and Tyr_493_ are highly conserved. Moreover, these conserved residues are involved in the interaction between the substrates and protein in subsites −1, +1, +2, and +3. We predict that Tyr_493_ plays an essential role by recognizing and cleaving the bond between the subsites −1 and +1, while the residues Gln_385_ and His_387_ form hydrogen bonds with the carboxyl groups in subsites +2 and +3, respectively. Furthermore, we propose that the subsites +1, +2, and +3 at the reducing end serve as a product releasing site. After the cleavage between −1 and +1, a trisaccharide is released into solution. Then, the substrate chain slides into the binding pocket of the catalytic domain to position the next trisaccharide for enzymatic cleavage [41].

## 4. Materials and Methods

### 4.1. Cloning, Expression and Purification of Full-Length and Truncated Aly7A

Full-length Aly7A (including the N-terminal CBM and the C-terminal catalytic module), its derivative CBM (N-terminal catalytic module only; residues 39–156) and Aly7A-CD (C-terminal catalytic module only; residues 246–506) were generated by PCR and ligated into the T7 expression vector pET-21a (+) (Novagen, Darmstadt, Germany). The recombinant plasmids were separately transformed in *E. coli* BL21 (DE3) which were grown at 37 °C in LB medium supplemented with 100 µg mL^−1^ ampicillin for 24 h. Enzyme was induced with 0.02 mM IPTG and incubation continued (20 °C, 20 h). Cells were centrifuged, resuspended in binding buffer and lysed by sonication at 4 °C. Binding and elution from a nickel nitrilotriacetic acid Sepharose (Ni-NTA Sepharose) column was performed according to the manufacturer’s instructions. Eluted proteins were subsequently analyzed by SDS-PAGE. A protein quantitative analysis kit was used for determination of protein concentrations (Beyotime Institute of Biotechnology, Nantong, China).

### 4.2. Enzymatic Activity Assay of Recombinant Aly7A and Aly7A-CD

Purified enzymes (0.1 mL) were mixed with 0.9 mL 20 mM Tris-HCl pH 8.0 containing 1% (w/v) sodium alginate and incubated at 30 °C for 10 min. The enzyme activity was determined by measuring the increased absorbance at 235 nm as previously described [45]. One unit of enzyme activity was defined as the amount of enzyme required to increase the absorbance at 235 nm by 0.01 per min.

### 4.3. Analysis of Aly7A and Aly7A-CDhydrolytic Patterns and Degradation Products

The reaction mixtures (800 μL) containing 1 µg of purified enzyme and 2 mg of substrate (sodium alginate, polyM, or polyG) were incubated at 30 °C for 0–48 h. The reaction mixtures were then treated as previously described [46] and then redissolved in 1 mL methanol. The hydrolysis products were then analyzed by thin layer chromatography (TLC) as previously described [46]. Then the products were separated by a fast protein liquid chromatography (FPLC). Using a Superdex Peptide 10/300 GL column (GE Healthcare, USA) to separate samples and monitor at wavelength 235 nm in an ÄKTA purifier system. The column was eluted with 0.2M NH_4_HCO_3_ at 0.5 mL/min as the flow rate. To further confirm the composition, the Aly7A final degradation products were centrifuged (12,000× *g*, 20 min) and 2 μL of supernatant was loop-injected onto an LTQ XL linear ion trap mass spectrometer (Thermo Fisher Scientific, Waltham, MA, USA). The samples were assayed in a positive-ion mode and the settings as previously reported [38].

### 4.4. Affinity electrophoresis of CBM

The binding activity of CBM towards alginate was evaluated by affinity electrophoresis according to the description previously reported [10,27]. The stacking gels contained 5% (*w/v*) polyacrylamide, and the separating gels contained 13% (*w/v*) polyacrylamide with or without 0.2% alginate. The purified CBM (3 µg) was loaded onto gels, and the gels were electrophoresed simultaneously for 2 h at 4 °C using a constant voltage of 120 V. Then, the gels were stained with Coomassie Blue for protein visualization.

### 4.5. Molecular Modeling and Docking Analysis

Protein Homology/analogY Recognition Engine V 2.0 was used for constructing the three-dimensional structure of Aly7A. The structure was based on the known structure of alginate lyase AlyB from *Vibrio splendidus*OU02 (PDB ID: 5ZU5), which has the 70% sequence similarity to Aly7A. Molecular docking analysis of Aly7A to alginate oligosaccharide ligand was performed using MOE (Molecular Operating Environment, Chemical Computing Group Inc., Montreal, Canada). The bound ligand in the crystal structure indicated the ligand binding sites. PyMOL (http://www.pymol.org) was used for analysis of modeled structure.

## 5. Conclusions

Herein, we investigated the degradation pattern of Aly7A by modular truncation and molecular docking. From our data, we predict that Aly7A adopts a unique action mode towards different types of substrates with the substrate chain sliding into the binding pocket of the catalytic domain to position the next trisaccharide for cleavage. In addition, we hypothesize that the carbohydrate binding module (CBM) and alpha-helical linker domains act together as a “controller” during the trisaccharide release process. This work has elucidated the role for a CBM as a regulator of product distribution and provides a new insight into a well-defined generation of alginate oligosaccharides with associated CBMs.

## Supplementary Materials

The following are available online athttps://www.mdpi.com/1660-3397/18/1/32/s1, Figure S1: SDS-PAGE analysis of purified Aly7A and Aly7A-CD. Lane M protein: protein marker; Lane 1: purified Aly7A; Lane 2: purified Aly7A-CD, Figure S2: FPLC analysis of Aly7A and Aly7A-CD final degrading products towards alginate, polyM and polyG. Table S1: Kinetic parameters of Aly7A and Aly7A-CD.
