# Supplementary material for: Elucidation of a Unique Pattern and the Role of Carbohydrate Binding Module of an Alginate Lyase
Source: Mar Drugs. 2019 Dec 30;18(1):32. doi: 10.3390/md18010032 (PMC7024192; doi:10.3390/md18010032)
Supplement: Supplementary file 1 [file marinedrugs-18-00032-s001.pdf]

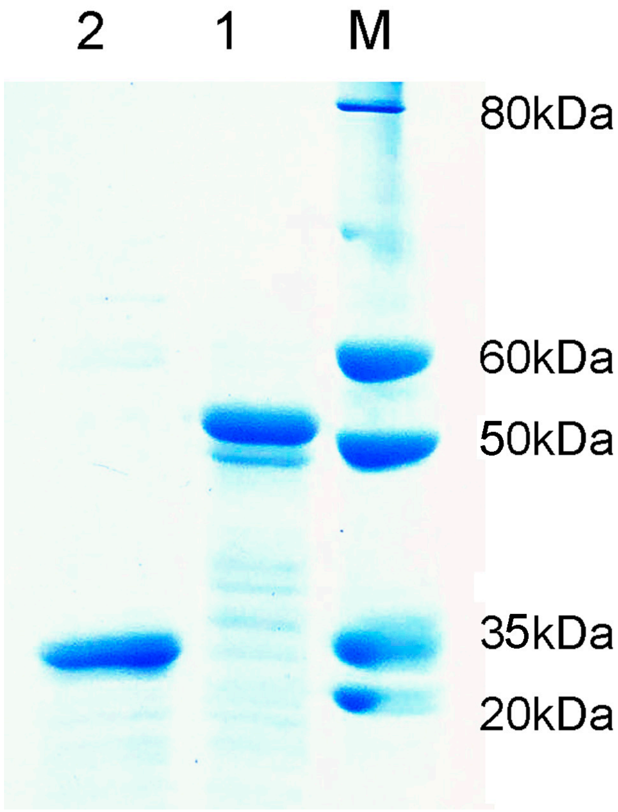

Fig. S1 SDS-PAGE analysis of purified Aly7A and Aly7A-CD. Lane M protein: protein marker; Lane 1: purified Aly7A; Lane 2: purified Aly7A-CD

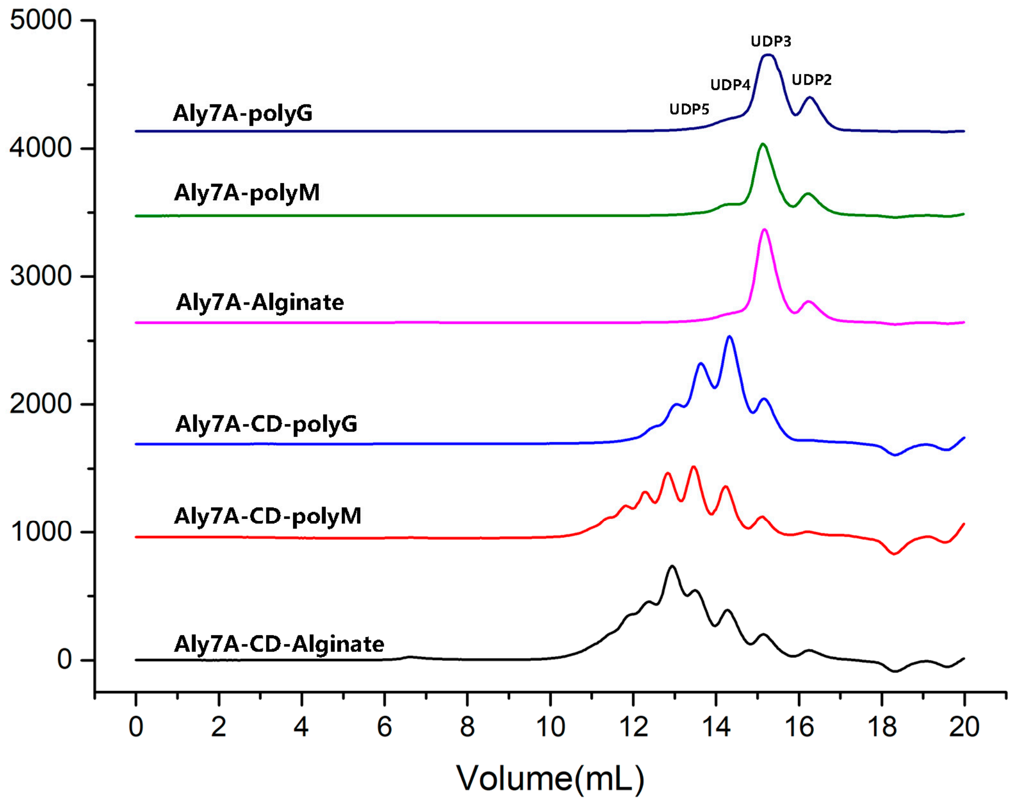

Fig. S2 FPLC analysis of Aly7A and Aly7A-CD final degrading products towards alginate, polyM and polyG.

Table S1. Kinetic parameters of Aly7A and Aly7A-CD

**Enzymes**	**Substrates**	V_{max}(μmol·s^{-1})	K_m(mM)	k_{cat} (s^{-1})	k_{cat}/K_m
	Alginate	0.22	0.23	17.3	75
Aly7A	polyM	0.23	0.40	17.6	44
	polyG	0.14	0.34	10.9	32
	Alginate	0.18	0.38	12.9	34
Aly7A-CD	polyM	0.17	0.36	12.1	33

	polyG	0.14	0.29	10.1	35
